# A novel gamma radiation-inactivated sabin-based polio vaccine

**DOI:** 10.1371/journal.pone.0228006

**Published:** 2020-01-30

**Authors:** Gregory J. Tobin, John K. Tobin, Elena K. Gaidamakova, Taralyn J. Wiggins, Ruth V. Bushnell, Wai-Ming Lee, Vera Y. Matrosova, Stephen J. Dollery, Heather N. Meeks, Diana Kouiavskaia, Konstantin Chumakov, Michael J. Daly

**Affiliations:** 1 Biological Mimetics, Inc., Frederick, MD, United States of America; 2 Department of Pathology, Uniformed Services University of the Health Sciences, Bethesda, MD, United States of America; 3 Henry M. Jackson Foundation for the Advancement of Military Medicine, Bethesda, MD, United States of America; 4 Defense Threat Reduction Agency, Ft. Belvoir, VA, United States of America; 5 Center for Biologics Evaluation and Research, Food and Drug Administration, Silver Spring, MD, United States of America; University of Adelaide, AUSTRALIA

## Abstract

A concerted action on the part of international agencies and national governments has resulted in the near-eradication of poliomyelitis. However, both the oral polio vaccine (OPV) and the inactivated polio vaccine (IPV) have deficiencies which make them suboptimal for use after global eradication. OPV is composed of attenuated Sabin strains and stimulates robust immunity, but may revert to neurovirulent forms in the intestine which can be shed and infect susceptible contacts. The majority of IPV products are manufactured using pathogenic strains inactivated with formalin. Upon eradication, the production of large quantities of pathogenic virus will present an increased biosecurity hazard. A logical ideal endgame vaccine would be an inactivated form of an attenuated strain that could afford protective immunity while safely producing larger numbers of doses per unit of virus stock than current vaccines. We report here the development of an ionizing radiation (IR)-inactivated Sabin-based vaccine using a reconstituted Mn-decapeptide (MDP) antioxidant complex derived from the radioresistant bacterium *Deinococcus radiodurans*. In bacteria, Mn^2+^-peptide antioxidants protect proteins from oxidative damage caused by extreme radiation exposure. Here we show for the first time, that MDP can protect immunogenic neutralizing epitopes in picornaviruses. MDP protects epitopes in Polio Virus 1 and 2 Sabin strains (PV1-S and PV2-S, respectively), but viral genomic RNA is not protected during supralethal irradiation. IR-inactivated Sabin viruses stimulated equivalent or improved neutralizing antibody responses in Wistar rats compared to the commercially used IPV products. Our approach reduces the biosecurity risk of the current PV vaccine production method by utilizing the Sabin strains instead of the wild type neurovirulent strains. Additionally, the IR-inactivation approach could provide a simpler, faster and less costly process for producing a more immunogenic IPV. Gamma-irradiation is a well-known method of virus inactivation and this vaccine approach could be adapted to any pathogen of interest.

## Introduction

After decades of coordinated effort, global campaigns to eradicate poliovirus (PV) may be nearing a successful conclusion with paralytic disease caused by wild viruses occurring in only three countries in the last two years [1–3] In the past 30 years, the global incidence of poliomyelitis disease has been reduced by >99.9% [1, 2]. The oral polio vaccine (OPV), composed of attenuated (Sabin) strains of the three serotypes, has been the workhorse of mass vaccination efforts because of its low cost, ease of administration, and ability to stimulate robust and durable immunity.

However, the attenuated OPV Sabin strains can revert to pathogenic phenotypes in vaccinated humans and, in rare cases, replicate chronically in individuals with primary immunodeficiencies. Circulating vaccine-derived polioviruses (cVDPVs) can cause poliomyelitis outbreaks in under-vaccinated and other susceptible populations [4–6]. With the eradication of wild type PV2, the trivalent OPV was replaced by bivalent OPV (bOPV; types 1 and 3) [7], lacking Sabin-2; and a similar strategy is expected for Sabin-3 now that wild type PV3 has been declared irradicated [8]. In 2013, the World Health Organization (WHO) mandated the introduction of inactivated polio vaccine (IPV) vaccination in countries with entirely OPV based vaccination programs. Eventually, the risk of vaccine-induced infections from OPVs will become more significant than the risk of wild virus infection; and ultimately, oral vaccines will be completely replaced worldwide [9].

Although IPV also protects from paralytic disease, it costs considerably more than OPV per dose and, unlike OPV, does not stimulate mucosal immunity in the gut [10]. Moreover, IPV manufacturing requires the production and handling of large quantities of pathogenic strains, with each fresh lot of wild type PV requiring many days of incubation in formalin at 37°C to ensure inactivation [11]. From a biosafety perspective, the use of live virus for vaccine production has resulted in outbreaks for polio and other viruses [12–14], and the WHO has cited vaccine production as “an important potential source of reintroduction” [15].

All of these factors can complicate the manufacturing process and exacerbate shortages. More than 20 countries have been facing considerable delays in the transition from OPV to IPV vaccination due to the limited supply of IPV over the past several years [16]. To aid vaccination programs, the Global Polio Eradication Initiative is pursuing strategies to generate more cost-effective and potent vaccines [17]. Although efforts to eliminate pathogenic viruses from the IPV manufacturing process by substitution with Sabin have resulted in vaccines licensed in Japan and China, concerns persist regarding the loss of potency caused by formalin damage to the dominant neutralizing epitope located in the B-C loop of VP1 of the prevailing PV1-Sabin strain [18, 19]. Therefore, finding a way to reliably inactivate the Sabin strains without damaging immunogenic properties is an important objective for the long-term continuation of polio vaccination during and after the global eradication of poliomyelitis.

We have developed a new method of preparing a Sabin IPV based on exposure to ionizing radiation (IR) in the presence of a Mn-decapeptide (MDP) antioxidant complex. The technology was adapted from mechanistic insights into the extreme radiation resistance phenotype of the bacterium *Deinococcus radiodurans* [20]. Manganous peptide complexes of *D*. *radiodurans* selectively protect proteins from reactive oxygen species (ROS), thereby preserving the functionality of cytoplasmic enzymes, including those needed to repair DNA. MDP is a synthetic analog of Mn^2+^-peptide-orthophosphate complexes that protect *D*. *radiodurans* from exposure to desiccation, ultraviolet C radiation, IR, and other forms of oxidative stress [21]. Specifically, MDP contains a rationally-designed decapeptide DEHGTAVMLK (DP1) which spontaneously forms a complex when DP1 and MnCl_2_ are combined in orthophosphate buffer [20]. MDP was previously demonstrated to be highly radioprotective of epitopes on Venezuelan equine encephalitis virus (VEEV) and Chikungunya virus when exposed to doses of IR (gamma rays) that irreversibly inactivate their genomes [20, 22]. Under aqueous conditions, MDP protects the structure and function of surface proteins exposed to massive IR doses (>40 kGy) but does not significantly protect DNA or RNA [22].

Gamma-irradiation is a well-studied virus-inactivation method and is also a Food and Drug Administration (FDA)-approved method for sterilizing medical devices, food, and household products [23, 24]. Although no gamma-irradiated vaccines have yet been licensed, manufacture of such vaccines has been implemented to the scale used in large phase III clinical trials [25]. During exposure to gamma radiation under aqueous conditions, the vast majority of oxidative damage to macromolecules is caused by the indirect effects of ROS produced by the radiolysis of water [21]. For irradiated whole-virus vaccines, IR-induced destruction of virus genomes is desired, while IR-induced damage to immunogenic epitopes on the surface of the virion must be prevented to preserve antigenic potency. A way to specifically protect epitopes from ROS-mediated damage during irradiation had not been possible until the discovery of *Deinococcus* Mn antioxidants [21, 26].

In this report, we show for the first time that MDP can protect crucial immunogenic proteins in two strains of picornaviruses (Sabin strains of PV1 and PV2 (PV1-S and PV2-S)), when exposed to doses of IR that fragment the viral genomes and inactivate infectivity. In contrast to formalin-inactivation of PV1-S, IR-inactivation in the presence of MDP causes substantially less damage to dominant neutralizing epitopes as suggested by the stimulation of higher titers of neutralizing antibodies in immunized rats. In the case of irradiated-PV2-S (IR-inactivated PV2-S), less viral protein is required to elicit equivalent immune responses compared to formalin-inactivated vaccines. The reduced mass of virus, when coupled to a streamlined and more rapid IR-inactivation process, could translate to a highly protective and less expensive inactivated Sabin-based IPV for increased safety in the post-eradication era. Our approach also offers a rapid response to other emerging enteroviruses, such as the virus implicated in acute flaccid myelitis [27].

## Materials and methods

### Cells and virus

H1-HeLa cells for virus production and MRC5 cells for virus titration and neutralization assays were purchased from American Type Culture Collection (ATCC, Manassas, VA). H1-HeLa cells were propagated by shaking suspension cultures in sMEM medium (Invitrogen, Thermo Fisher Scientific, Waltham, MA) supplemented with 10% newborn calf serum. MRC5 cells were propagated as monolayers in MEM medium (Invitrogen, Thermo Fisher Scientific, Waltham, MA) supplemented with 10% fetal bovine serum. Although the majority of virus for commercial IPV manufacturing is produced on adherent Vero cell derivatives, H1-HeLa cells were selected for proof of concept due to ease of use.

Attenuated Sabin strains of PV1 and PV2 were purchased from ATCC (Manassas, VA), propagated in H1-HeLa cells, and purified using sucrose density gradients. Briefly, H1-HeLa cells were concentrated to 4 × 10^7^ cells/mL, incubated 1 h at 25°C with virus at a concentration of 10 CCID_50_ per cell, diluted to 4 × 10^6^ cells/mL, and shaken at 37°C. Approximately 7 to 9 h after incubation, the cells were pelleted at 500 × *g* for 5 min and resuspended in phosphate buffered saline (PBS) containing 0.1% (w/v) NP40 detergent to release progeny virions. The cell lysate was clarified by centrifugation at 10,000 × *g* for 10 min. The virus was purified in a two-step ultracentrifugation process. In the first step, the clarified cell lysates were layered over 30% (w/v) sucrose in PBS and pelleted at 120,000 × *g* for 7 h at 16°C in a Beckman SW-28 rotor. Pellets were resuspended in PBS and banded by centrifugation at 120,000 × *g* for 4 h at 16°C in a 7.5–45% linear sucrose gradient in PBS. 0.01% bovine serum albumin was added as a carrier protein. The virus band (at approximately 30% sucrose) was harvested from the gradients and frozen in aliquots. Virion concentration was determined by OD_260_ using the extinction coefficient 1 OD = 0.133 mg/ml.

Control vaccines, IPOL (manufactured by Sanofi Pasteur Inc., Swiftwater, PA) was purchased from Moore Medical LLC (Farmington, CT) and VeroPol was purchased directly from the manufacturer, Staten Serum Institut (Copenhagen, Denmark).

### Virus infectivity assay

Standard virus infectivity assays were performed in 48-well plates of MRC5 cells. When the cells were at 80% confluence, media in 4 or 6 replicate wells was replaced with 50 μl aliquots of each 10-fold virus dilution. Plates were incubated at 25°C for 1 h to allow virus attachment. After attachment, 0.5 mL of medium was added to each well and the plates were incubated for 4–6 days at 37°C. Wells were scored as infected or uninfected by microscopic visualization of cytopathic effects (CPE) due to virus infection. Titers were determined using the Spearman-Kärber formula (log_10_ 50% endpoint dilution = —(x_0_ - d/2 + d ∑ r_i_/n_i_) where x_0_ = log_10_ of the reciprocal of the highest dilution (lowest concentration) at which all wells are positive; d = log_10_ of the dilution factor; n_i_ = number of wells used for each individual dilution; r_i_ = number of positive wells) [28–30].

To determine whether virus infectivity survived irradiation at 35, 40 and 45 kGy for PV1-S, and 40, 45, and 50 kGy for PV2-S, the samples were adjusted to 1 mL and 50 μl aliquots of virus were applied to MRC5 cells in 24-well plates for 1 h at 25°C. After the virus attachment phase, 1 mL of medium was added to each well and the plates were incubated for 4 days at 37°C. Plates were examined microscopically for the presence of cytopathogenic effect (CPE) and titers, if any, calculated using the Spearman-Kärber formula as above. To confirm the lack of infectivity in virus irradiated at higher doses of gamma radiation, potential low levels of virus were released from the cytoplasm by freeze-thaw and used to inoculate a second round of test plates. The process was repeated through five rounds of sequential passage to ensure that no trace infectivity remained in the irradiated sample.

### Virus irradiation

PV1-S and PV2-S were compounded in 25 mM potassium phosphate buffer, pH 7.4 at a concentration of 0.08–0.3 mg/mL virus using varying concentrations of MnCl_2_ and the decapeptide (DP1: DEHGTAVMLK). Pilot studies determined optimal concentrations of 0.2 mg/mL virus, 3 mM MnCl_2_ and 3 mM DP1 for maximum preservation of viral proteins as assessed by semi-quantitative immunoblotting. Stabilized virus samples were placed into 0.5 mL skirted o-ring tubes (Denville Scientific, Inc., Holliston, MA.). Ambient air within the tubes was replaced with argon (inert gas) to reduce oxidation. The tubes were closed tightly, sealed with parafilm, and placed on wet ice. Samples were irradiated in a ^60^Co irradiation unit at 10 kGy/h on wet ice for varying lengths of time. For example, 4.5 h of exposure was required to deliver 45 kGy gamma irradiation. Samples were then stored at -80°C until ready for analysis.

### D antigen ELISA

Poliovirus D-antigen ELISAs were performed to determine the level of neutralizing epitopes present in inactivated poliovirus vaccine preparations as previously described [31]. In brief, rabbit polyclonal antibodies against poliovirus serotypes were coated on ELISA plates and incubated overnight at 4°C. Plates were then washed in PBS containing 0.05% Tween 80 and blocked in PBS containing 3% rabbit serum for 1 h at RT. Inactivated vaccines and reference standards were captured overnight at 4°C. Plates were washed and incubated with biotinylated detection antibody for 1h at 37°C. Plates were again washed and incubated with ExtrAvidin conjugated horseradish peroxidase for 45 min at RT. Plates were washed and incubated with 3,3',5,5'-tetramethylbenzidine substrate and the reaction stopped by the addition of sulfuric acid. Plates were read at 450 nm and values calculated using linear regression.

### Western blot analysis

Irradiated viruses were analyzed for protein preservation using semi-quantitative western blot assays. Samples of irradiated virus were denatured and electrophoresed using mini-protean TGX denaturing polyacrylamide gels (Bio-Rad Laboratories Inc., Hercules, CA) and then electrotransferred to nitrocellulose membranes using the BioRad Turbo Transfer device and Transfer kit as per the manufacturer’s recommendations. The membranes were incubated for 2 h or overnight in a solution of 10% non-fat dried milk in PBS, reacted with goat anti-PV1,2,3 polyclonal antibody (#PA1-73124; Invitrogen, Thermo Fisher Scientific Inc., Waltham, MA) at 1:1000 dilution in 2% non-fat dried milk solution for 1 h at room temperature and washed approximately 10 times in 1 × Tris buffered saline with 0.1% Tween-20 for 6 min each. The blots were then probed with rabbit anti-goat antibody conjugated to horseradish peroxidase (1:1000 dilution) in 2% non-fat dried milk solution; #14-13-06; KPL Inc., SeraCare Life Sciences, Milford, MA), and developed with Clarity Western ECL substrate (Bio-Rad Laboratories Inc., Hercules, CA) as per the manufacturer’s protocol. ECL signal was detected using standard x-ray film. Densitometry analyses were performed to compare signal strength among samples.

### PCR based detection of RNA fragmentation

For assessment of RNA fragmentation, viral RNA was extracted from 10–20 μl of irradiated virus samples using phenol and then precipitated with isopropanol. The RNA pellet was washed twice with 75% ethanol, air-dried and then dissolved in 20 μl water. cDNA was synthesized from 20 μl (2–3 μg) viral RNA (50 μl reactions) with an oligo-dT primer and Promega ImProm-II reverse transcriptase. An aliquot (4 μl) of cDNA was used in a 20 μl PCR reaction (94°C for 2 min followed by 16 cycles at 52°C for 30 s and 68°C for 50 s with a final extension at 68°C for 7 min) containing the 3' and 5' primer sets. The 3' primer set amplifies a 617 bp region corresponding to nt 6786–7403 and the 5' primer set amplifies a 463 bp region corresponding to nt 5478–5941. The primers were selected to amplify two fragments near the 3’ end of the genomic RNA that could be resolved by standard agarose electrophoresis. A key feature of the fragment design is that the 617 bp fragment resides immediately adjacent to the oligo(dT) priming site and the 463 bp fragment resides further away from the cDNA priming site. Thus, random fragmentation of the genome will reduce the intensity of the smaller fragment prior to reduction of the intensity of the larger fragment. The resulting PCR products were resolved by electrophoresis in a 1.2% agarose and then visualized with SYBR Safe DNA Gel Stain (Thermo Fisher Scientific, Waltham, MA).

### Transmission electron microscopy

Approximately 5 μl volumes of each formaldehyde-fixed poliovirus sample were placed on carbon-stabilized, formvar-coated 400-mesh copper grids (Electron Microscopy Sciences, Hatfield, PA) for 5 min. Thereafter, the grids were gently washed with water followed by addition of 5 μl aqueous 2% uranyl acetate for 1 min. The grids were then wicked off, air-dried and examined in a JEOL JEM-1011 transmission electron microscope (JEOL USA, Inc., Peabody, MA) and images were recorded on an AMT XR50 digital camera (Advanced Microscopy Techniques, Corp. of Woburn, MA). Virion particles that closely resembled unirradiated virus were counted by visual (manual) enumeration of virion images on multiple micrographs. Virion particles that more closely resembled broken or empty particles were also counted by visual enumeration of virus images. In each sample >700 particles were analyzed and counted.

### Rat immunization

The animal studies were reviewed and approved by the Animal Care and Use Review Office (ACURO) of the U.S. Army Medical Research and Development Command (USAMRDC) and the Institutional Animal Care and Use Committee (I-ACUC) of Sobran Bioservices, Inc. All rat studies were performed at Sobran Bioservices, Inc. (Baltimore, MD) under humane conditions by trained personnel. Wistar rats, a widely accepted animal model for polio vaccine analysis [29, 32], between 6 and 8 weeks of age and of mixed sex were used for immunization studies. A total of 180 rats were used in these studies. Animals were randomized and assigned to groups of 4 or 8 prior to immunization. Figs 4, 5 and S3 report the number of rats in each group. The rats were housed in a pathogen-free facility with *ad libitum* access to food and water in standard isolator cages with 2–3 rats per cage. Animals were observed twice daily to assess potential health problems. No differences in health observations, weight, or behavior were detected between immunization groups before or after immunization. No adverse events were observed from the immunizations. After a 1-week acclimation, the rats were immunized by intramuscular injection into quadriceps without adjuvant (Day 1) and then boosted three weeks later (Day 21). Serum samples were prepared from coagulated whole blood on Days 35 and 49. For the interim bleeds taken on Day 35, the animals were gently restrained and approximately 0.2 mL of whole blood was withdrawn from the saphenous vein. For final bleeds taken on Day 49, the animals were anesthetized with pentobarbital and approximately 2 mL of blood was withdrawn through a cardiac puncture prior to exsanguination with a second dose of pentobarbital.

### Virus neutralization assay

End-point neutralization titers were determined to compare the immunogenicity of irradiated polioviruses with formalin-fixed conventional IPV. Serial two-fold dilutions of serum were incubated 1 h with 100 CCID_50_ of PV1-S or PV2-S. The virus-serum mixtures were applied to 4 or 6 replicate wells in 48-well plates of MRC5 monolayers at room temperature. After a 1 h incubation, unbound virus was removed by one wash with 0.5 mL PBS, 0.5 mL complete media was added to each well, and the plates were incubated for 4–6 days at 37°C. Wells were scored as infected or uninfected by microscopic visualization of CPE. Neutralization titers were derived using the Spearman-Kärber formula [28–30]. The titer represents the reciprocal of the highest dilution of serum that causes a 50% reduction in the number of infected wells. The neutralization titers were graphed as Log_2_ values.

### Decapeptide ELISA

In order to confirm that the peptide within the MDP complex is not immunogenic, we evaluated antibody responses against the MDP complex. All incubations were performed at room temperature (approximately 27°C). 96-well plates were coated overnight with 5 nanograms of peptide per well in a volume of 100 microliters of water. Following four washes with 1 × PBS-T (phosphate buffered saline with 0.1% Tween-20), non-specific sites in the wells were blocked using a 2-h incubation using a solution of 10% non-fat dried milk in PBS-T and the wells were probed in quadruplicate with 1:500 and 1:1500 dilutions of test rat sera. After 2 h, the plates were washed four times with 1 × PBS-T and incubated 1 h with horseradish peroxidase-conjugated goat anti-rat IgG (Kierkegaard and Perry, Gaithersburg, MD). Plates were washed with 1 × PBS-T and developed using the SureBlue Reserve peroxidase substrate (SeraCare Life Sciences, Milford, MA). The reactions were stopped with the addition of 0.1N HCl. The colorimetric development was quantitated using an ELISA plate reader at OD_600_. Mean absorbance values and their standard deviations were graphed.

### Statistical analysis

In order to evaluate the immunogenicity of irradiated vaccines and commercially prepared IPV vaccines, unpaired, parametric, one-tailed Student’s t tests were performed. Neutralizing titers stimulated by irradiated vaccine samples were compared to neutralizing titers stimulated by either IPOL or VeroPol independently to test the hypothesis that the irradiated vaccines are more immunogenic than either of the commercially prepared vaccines used in comparison. Analyses were performed using GraphPad, Prism version 8.2.1. P values are reported within the figures.

## Results

### Poliovirus is inactivated by gamma-irradiation in the presence of MDP

PV1-S and PV2-S virus stocks used in this study were propagated in mammalian cell suspension cultures and purified to approximately 95% purity using sucrose density gradients [33]. To confirm the high quality and purity of both PV1-S and PV2-S stocks, viral proteins were resolved on SDS-PAGE and stained with Coomassie. The three major capsid proteins, VP1, VP2, and VP3 were distinctly visible between 40–25 kDa (S1 Fig). The smallest capsid protein, VP4 (~7.4 kDa), was not visible. The purified PV1-S and PV2-S stocks were then used for inactivation studies by exposure to different doses of gamma radiation with or without the MDP complex.

**Fig 1:**
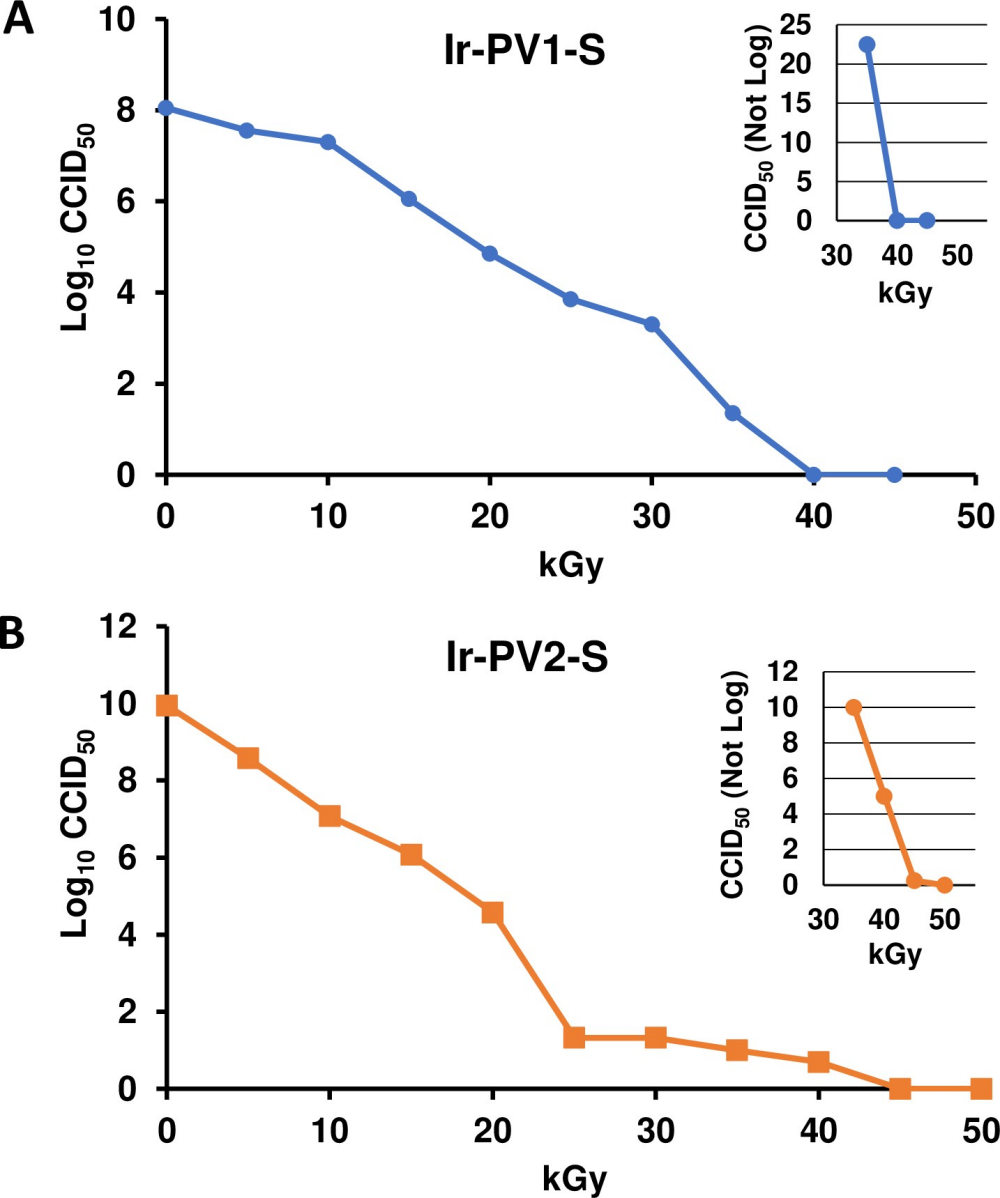
Reduction in infectivity of gamma-irradiated PV. **A**) PV1-S and **B**) PV2-S samples combined with the MDP complex were exposed to increasing doses of gamma radiation and tested for infectivity using a CCID_50_ assay. The larger graphs report the Log_10_ values, the insets report non-log titers showing no infectivity at 40 and 45 kGy for PV1-S; and 45 and 50 kGy for PV2-S. Data shown are representative of over five independent experiments. Ir-PV1-S and Ir-PV2-S, gamma-irradiated PV-S.

A series of irradiation experiments were performed to identify the optimal sample preparation conditions using western blot analysis as an approximate gauge of the protection of viral capsid proteins from IR damage. The optimal sample composition was found to be 3 mM decapeptide, 3 mM MnCl_2_, 25 mM potassium phosphate buffer (pH 7.4) and >160 μg/mL virion (total protein). Various radiation exposure doses (0 kGy– 50 kGy) were evaluated to identify the minimum dose of gamma radiation required to completely inactivate the virus in the presence or absence of the MDP complex. In order to evaluate virus inactivation upon irradiation, a standard cell culture infectivity assay (CCID_50_) was used. Fig 1A and 1B demonstrate the loss of infectivity as a function of increasing doses of gamma radiation for PV1-S and PV2-S. For PV1-S samples exposed up to 45 kGy and PV2-S samples exposed up to 50 kGy, the amount of virus infectivity remaining was evaluated using the standard titration assay (graphed as Log_10_ CCID_50_ per mL of irradiated virus). To analyze residual, low-level infectivity in the samples exposed to higher doses of radiation, we scaled-up amounts of virus to 16 μg virus in 0.5 mL and plated samples directly onto cell monolayers without dilution. To confirm the absence of infectious virus in samples exposed to the highest doses of radiation, freeze-thaw lysates from wells with no visible cytopathic effect (CPE) were passaged on fresh cell monolayers an additional four cycles. Fig 1A and 1B show samples of PV1-S and PV2-S, exposed to a range of IR doses, respectively. The inserts in Fig 1A and 1B (top right) shows the linear CCID_50_ values from samples exposed to 35, 40 and 45 kGy for PV1-S, and 40, 45, and 50 kGy for PV2-S. For example, the PV1-S sample exposed to 35 kGy retained 22 infectious units, whereas PV2-S exposed to 40 kGy retained 4 infectious units in a sample containing 16 μg of virus. No infectivity was detected in the PV1-S samples exposed to 40 kGy or higher, or in the PV2-S samples exposed to 45 kGy or higher. The difference in the radiation dose resulting in complete inactivation of the two viruses may be explained by the almost 100-fold increase in specific infectivity per volume of PV2-S compared with PV1-S (Fig 1).

Because 45 kGy is the minimum dose of gamma rays that quantitatively and reliably abolished infectivity, this dose was chosen to prepare virus for immunization and other studies.

### MDP protects the proteins of poliovirus from oxidative damage but not the genome

**Fig 2:**
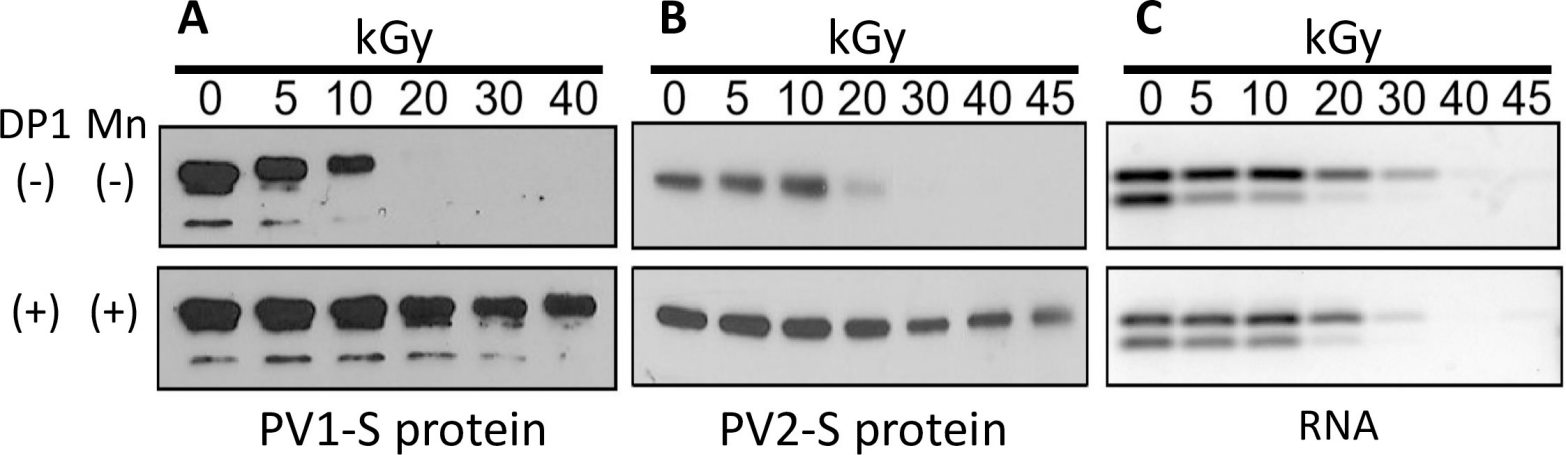
Analysis of irradiated PV. **A**) and **B**) show western blot analysis of PV1-S and PV2-S samples (respectively) that were irradiated at the indicated doses of gamma radiation in the presence (+) or absence (-) of DP1 and MnCl_2_ (Mn) as shown. **C)** Electrophoresis of PCR products from PV2-S samples irradiated for the times indicated, +/- MDP. Viral RNA was extracted from samples following treatments and subject to RT-PCR. RT-PCR products were detected with a subsequent round of PCR and the resulting DNA fragments were resolved via electrophoresis and visualized using SYBR Safe DNA Gel Stain. DP1, decapeptide 1: DEHGTAVMLK.

To determine if PV1-S and PV2-S proteins could be protected by the MDP complex during supralethal irradiation, samples were irradiated in the presence and absence of MDP and western blot analyses were performed using anti-capsid polyclonal antibody (Fig 2A, PV1-S, and Fig 2B, PV2-S). The presence of the MDP complex (DP1 plus MnCl_2_) protected the viral capsid proteins from radiation-induced oxidative damage. When one or more components of the MDP complex i.e., the DP1 or MnCl_2_, were omitted from the sample composition, the capsid proteins were more vulnerable to radiation damage at the doses tested in our study (Fig 2A and 2B and S2 Fig).

Following the observation that PV proteins are protected by MDP, the protection of the viral RNA genome was examined using a PCR-based fragmentation assay (Fig 2C and S2 Fig). Viral RNA was purified from PV2-S samples exposed to varying doses of gamma radiation in the presence and absence of MDP. cDNA of purified genomes and fragments was synthesized using oligo(dT) primers that anneal to the poly(A) sequence at the 3’ end of the viral RNA. Subsequent PCR was performed on the resulting templates using two primer sets located at different distances from the 3’ end. The upper bands (Fig 2C) represent amplicons immediately adjacent to the 3’ genomic cDNA and the lower bands represent amplicons approximately 1500 nt upstream. Reductions in the intensity of the PCR bands indicate increased fragmentation of the RNA. As expected, the viral genome was significantly degraded and cleaved after radiation exposure in a dose-dependent manner. The intensity of the lower (upstream) band was reduced at lower doses of radiation in comparison to the upper bands due to the fact that a longer stretch of intact viral genome is required to generate the amplicon. The greater the distance from the poly(A) tail that an amplicon is located, the greater the likelihood that IR-induced cleavage will have occurred and cDNA strand synthesis will terminate before the amplicon. Equivalent PCR band intensities were observed irrespective of the presence or absence of MDP (or individual components of the complex, DP1 or MnCl_2_ (Fig 2C, top and bottom panels). This indicates that MPD does not interfere with radiation-induced genome degradation. The two PCR amplicons generated in the assay were used to correlate the inactivation of viral infectivity with single strand break (SSB) damage in the viral genomic RNA [34]. The observed genome degradation correlated with the loss of infectivity seen in the CCID_50_ assay (Fig 1).

### Electron microscopic evaluation of inactivated poliovirus

**Fig 3:**
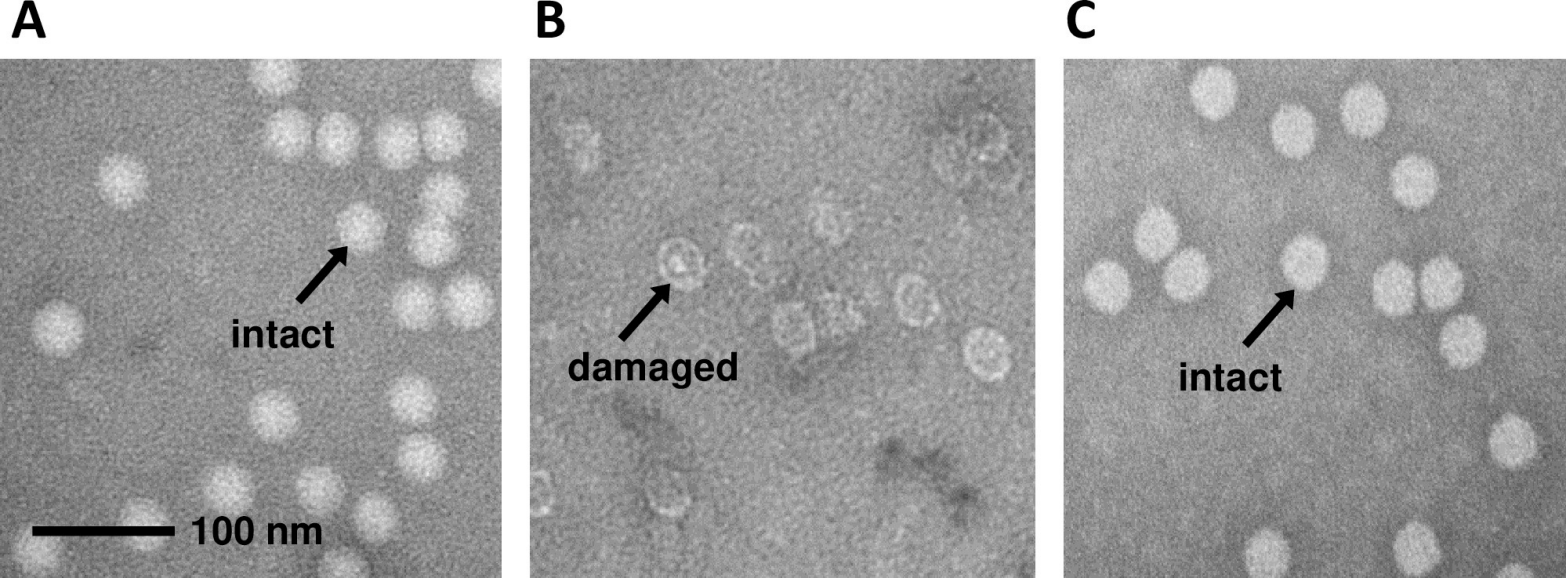
Ultrastructural analysis of irradiated poliovirus. Electron micrographs are shown of PV1-S that had been **A**) not irradiated, **B**) irradiated to 45 kGy without MDP, or **C**) irradiated to 45 kGy in the presence of MDP. The images are representative of >700 particles assayed for each group. The length of the scale bar in Panel A is 100 nm.

Although the MDP complex protected viral proteins from oxidation and radiation-induced damage, we also wanted to assess whether viral particles retained their overall structure after radiation exposure in the presence of MDP. Ultrastructural analyses were performed by transmission electron microscopy of PV1-S particles in samples that were either not irradiated (Fig 3A), irradiated (Fig 3B), or irradiated in the presence of MDP (Fig 3C). Virus particles exposed to gamma radiation (45 kGy) in the presence of the MDP complex appeared very similar to unirradiated virus particles in morphology and density. In contrast, PV irradiated (45 kGy) without the MDP complex showed fragmentation with hollow-looking irregular particles. Electron micrographs were examined visually to count the number of particles that were similar in appearance to unirradiated virus and the number of particles that appeared to be damaged. Upon evaluation, 91.8% of the non-irradiated particles appeared to be high-density, intact particles. In comparison, 90.0% and 2.0% were high-density particles in samples irradiated with and without MDP, respectively (as determined by analysis of >700 particles per sample). These observations are consistent with the protein protection data (Fig 2) and demonstrate the protective potential of the MDP complex on overall particle morphology.

### Irradiated poliovirus vaccine candidates are highly immunogenic

After delineating conditions under which poliovirus is completely inactivated but the proteins appear protected, we evaluated the immunogenic potential of irradiated PV as a vaccine candidate. The stimulation of neutralizing antibodies in Wistar rats is a widely-accepted test of vaccine potency [35]. Groups of four or eight rats were immunized intramuscularly on Days 1 and 21 with irradiated PV1-S or PV2-S (Ir-PV1-S, Ir-PV2-S) or two control vaccines, IPOL (Sanofi Pasteur) and VeroPol (Staten Serum Institute) via intramuscular injection without adjuvant. A group of eight rats were immunized with PBS as a mock antigen control. Prior to immunization, irradiated PV samples were assayed for D-antigen unit (DU) content to normalize the antigen dose to a standard human dose of vaccine [31]. Animals were immunized with full or fractional doses with one dose defined as containing 40 DU of PV1-S, and 8 DU of PV2-S. Sera collected on Day 49 for PV1-S, and Day 35 and 49 for PV2-S were assayed for neutralization antibody responses against PV1-S or PV2-S respectively. Although many *in vivo* assays of this nature focus on the number of animals that seroconvert to a titer of 1:8, we have used extinction serum neutralization titers to compare the immunogenicity of the irradiated and formalin-inactivated viruses more finely.

**Fig 4:**
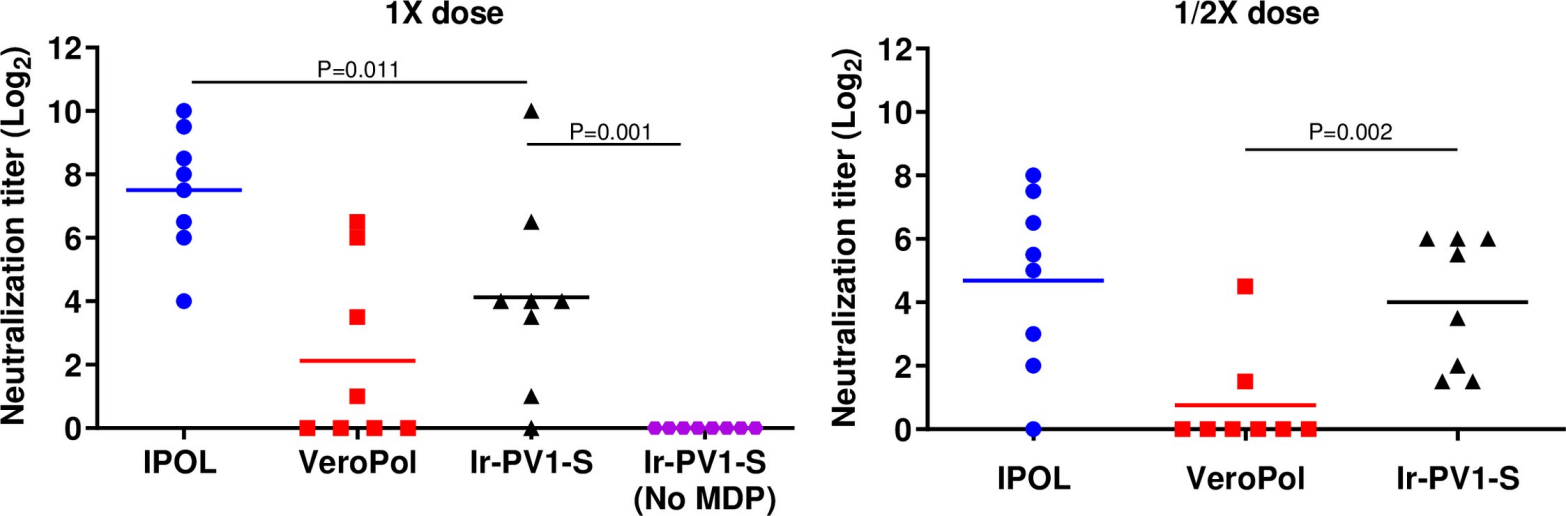
Neutralizing antibody response against PV1-S. Anti-PV1 neutralization titers from groups of 8 rats immunized with irradiated 1 × or 1/2 × human dose of IPOL (blue circle), VeroPol (red square), IR-inactivated PV1-S (black triangle) or irradiated PV1-S without MDP (purple circle) are shown here. Neutralization titers for D49 are presented as Log_2_ values. Sera from rats immunized with a saline control had titers below the limit of detection. Horizontal lines indicate mean neutralization values for each group and each data point represents an individual animal within the group. P values from unpaired one-tailed t-tests comparing Ir-PV1-S to either IPOL or VeroPol are indicated. P values above 0.05 are not shown. Ir-PV1-S, gamma-irradiated PV1-S. The results reflect serological analyses from the sera from one animal immunization study.

Fig 4 reports the titers from rats immunized with 1X dose (40 DU) of PV1-S. Sera from rats immunized with IPOL and VeroPol had mean Log_2_ neutralization titers of 2^7.5^ and 2^2.1^ at day 49, respectively. Sera from rats immunized with IR-inactivated PV1-S had titers of 2^4.1^. Sera raised against PV1-S that had been irradiated without the MDP complex resulted in no detectable neutralization titers. A half-dose (1/2 ×, 20 DU) of IR-inactivated PV1-S stimulated neutralization titers which were higher than those from the same dose of VeroPol and slightly lower from those of the half dose of IPOL (Fig 4). This may be an important first step in increasing the number of vaccine units that can be produced per batch of PV.

**Fig 5:**
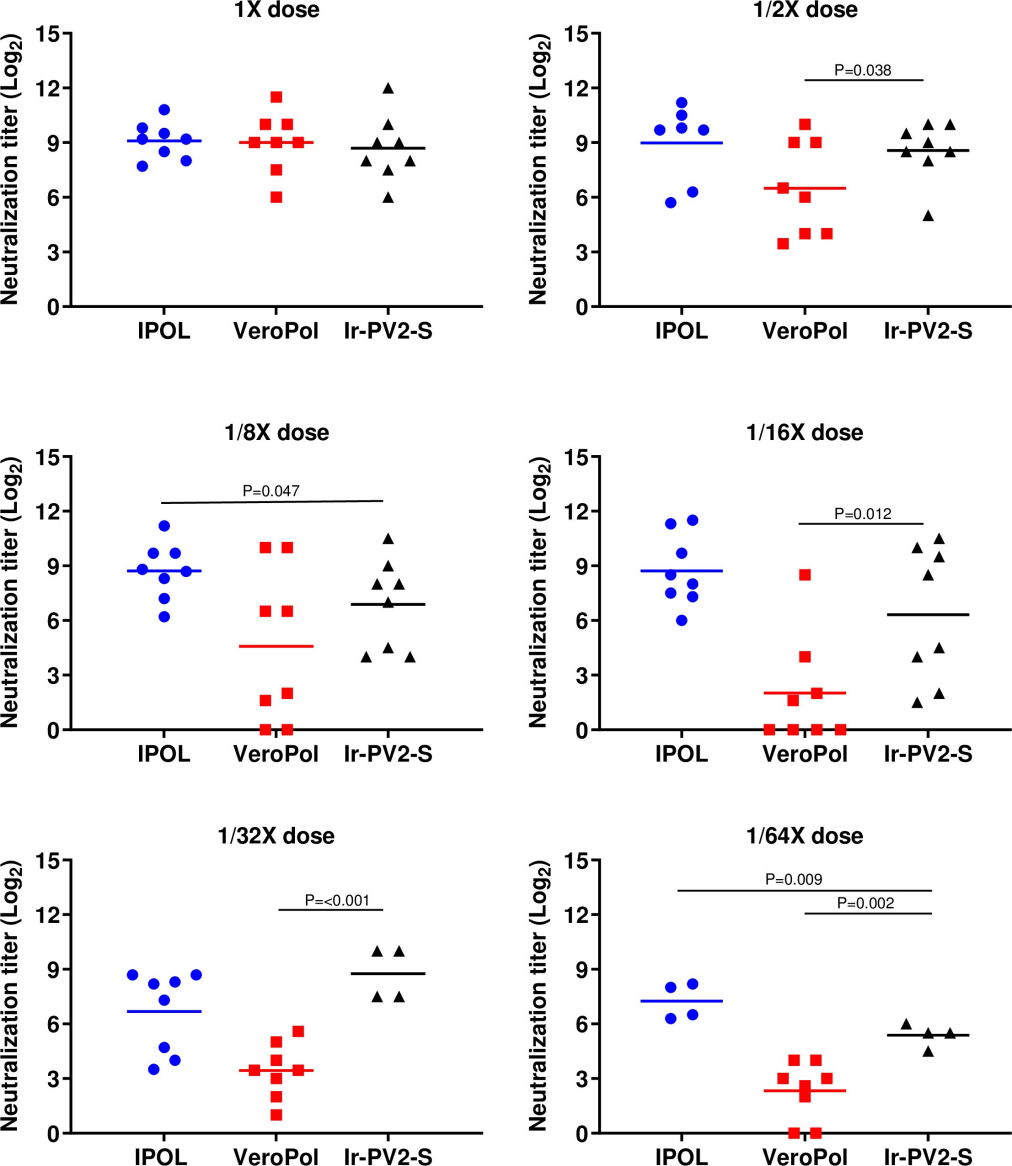
Neutralizing antibody response against IR-inactivated PV2-S. Anti-PV2 neutralization titers from groups of 8 rats immunized with 1 ×, 1/2 ×, 1/8 ×, 1/16 ×, 1/32 × or 1/64 × human doses of IPOL (blue), VeroPol (red), or IR-inactivated PV2-S (black). are shown here as indicated. Neutralization titers for D49 are presented as Log_2_ values. Horizontal lines indicate mean neutralization values for each group and each data point represents an individual animal within the group. Ir-PV2-S, gamma-irradiated PV2-S. P-values from unpaired one-tailed t-tests comparing Ir-PV2-S to either IPOL or VeroPol are indicated. P-values above 0.05 are not shown. The results reflect serological analyses from the sera from one animal immunization study.

Analogous studies were performed using irradiated PV2-Sabin (Ir-PV2-S). Anti-PV2-S neutralization titers for each animal immunized with different human doses (1 × 8 DU of PV2-S down to 1/64 × dose) are shown for days 49 and 35, respectively (Fig 5 and S3 Fig). All three vaccine preparations resulted in similar levels of neutralizing titers at 1 × dose on D49 i.e., 2^9.1^, 2^9.0^ and 2^8.7^ for IPOL, VeroPol and Ir-PV2-S respectively. However, neutralization titers declined more substantially for VeroPol starting at 1/2 × dose (2^6.5^ at 1/2 ×, 2^4.5^ at 1/8 ×, 2^1.9^ at 1/16 × dose, 2^3.2^ at 1/32 × dose, and 2^2.3^ at 1/64 × dose), whereas IPOL and Ir-PV2-S maintained higher neutralizing titers (i.e. 2^9.0^ and 2^8.5^ at 1/2 × dose, and 2^7.2^ and 2^5.4^ at 1/64 × dose respectively) (Fig 5). The percentage of rats that seroconverted (i.e., resulted in a neutralizing titer of ≥2^3^) was higher with the IR-inactivated PV2-S than VeroPol for all fractional doses. Similar trends were observed at both D49 and D35 (Fig 5 and S3 Fig).

### MDP complex is not immunogenic

**Fig 6:**
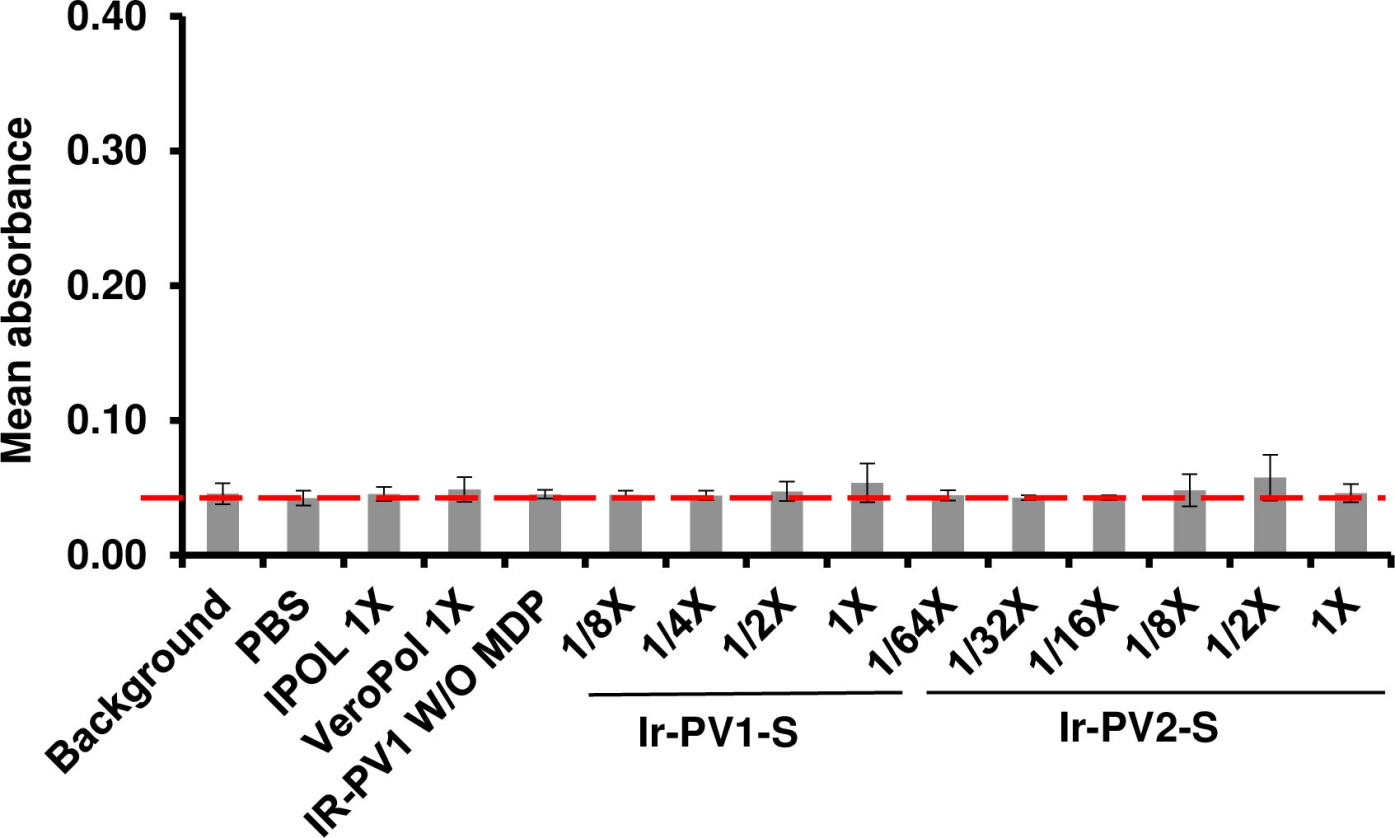
ELISA analysis of Wistar rat sera for MDP peptide reactivity. Sera from immunized rats were tested for anti-peptide antibodies using 96-well plates, which were coated with 5 ng DP1. Mean absorbance values of quadruplicate wells were plotted with standard deviations (mean values from 8 biological replicates per group). Values from wells probed without sera (Background or baseline, indicated with a red dotted line) or sera from rats immunized with either PBS, a 1 × human dose of IPOL, VeroPol, or IR-inactivated PV1-S without MDP were included as controls. Mean absorbance values from wells probed with sera from Wistar rats immunized with indicated doses of PV1-S and PV2-S are shown here. No significant differences were observed between any of the groups tested above. Ir-PV1-S and Ir-PV2-S, gamma irradiated PV-S.

In order to confirm that the peptide within the MDP complex is not immunogenic, we evaluated antibody responses against the MDP complex in the rats immunized with the inactivated vaccines. 96-well plates were coated with the decapeptide used in the MDP complex and probed with 1:500 and 1:1500 dilutions of the rat sera. ELISA binding and wash buffers did not include 3mM MnCl_2_ as we have shown previously that this does not alter the conformation of the decapeptide and may alter the biochemistry of the ELISA [36]. The assay was conducted with sera from various doses of IR-inactivated PV1-S and IR-inactivated PV2-S as shown (Fig 6). Negative controls included sera from rats immunized with either 1 × PBS, 1 × human dose of IPOL or VeroPol and PV1-S irradiated without MDP. None of the serum samples showed significant binding to the decapeptide indicating that the complex does not result in antibody responses to the peptide.

## Discussion

To maintain a polio-free world after eradication, all nations will need to remain vigilant by continuing surveillance and vaccination for an indeterminate number of years. Additionally, the containment of any live or wild type virus will be of enormous importance. As part of the Global Polio Eradication Initiative, the WHO has developed procedures to be followed by countries retaining wild type polioviruses for IPV production to minimize the risk of accidental release of neuropathogenic viruses into the environment [4, 9]. Substitution of attenuated Sabin strains in the manufacturing process for IPV would reduce the biohazard risks substantially [37]. Recently, Sabin-based IPV products have been licensed for use in Japan and China. However, the formalin-inactivation process has been shown to inactivate neutralizing epitopes in the Sabin strains [19, 37, 38]. For example, formalin mediates damage to the immunodominant epitope (site 1) in the B-C loop of vp1 for PV1-Sabin by reacting with a lysine residue [39]. The use of irradiation for inactivation circumvents this potential complication and also results in a highly simplified production process compared to formalin inactivation. Additionally, the preservation of immunogenic epitopes may allow an increase in the number of doses per milligram of the inactivated vaccine preparation, thus leading to potential reductions in virus mass per dose. The production of larger quantities of doses per milligram of pure virus and a streamlined inactivation process could mitigate periodic shortages of available vaccine [16].

Gamma irradiation is an FDA-approved method of sterilization for multiple medical, surgical and dental devices [39, 40]. Gamma irradiation results in direct and indirect damage to proteins, lipids, and nucleic acids that can cause loss of cellular or viral viability. Generally, the probability that sparsely ionizing radiation will result in lethal damage is directly correlated to the size of the genome. As a rule, far higher doses of radiation (e.g., 20–45 kGy) are required to inactivate viruses as compared to bacteria or mammalian cells because of disparities in genome sizes. Surface epitopes that convey protective immunity are destroyed at supralethal doses unless they are specifically protected. For these reasons, no irradiation-inactivated vaccines for virus pathogens have been approved for human use as the irradiation process destroys neutralizing epitopes unless protective agents, such as MDP, are included at the time of irradiation [23, 41, 42].

An ideal irradiated vaccine development approach will seek to protect immunogenic proteins from the oxidative damages caused by supralethal irradiation without compromising DNA damage. The MDP-irradiation approach described herein is a feasible solution to the limitations normally imposed by the use of supralethal irradiation. Our study clearly demonstrates complete inactivation of PV preparations by IR doses of 45 kGy while protecting the surface epitopes required for high immunogenicity upon vaccination. The data indicate that the MDP complex protects PV surface proteins responsible for inducing strong neutralizing antibody responses, even at the highest IR dose tested (45 kGy). In contrast, complete degradation of the surface epitopes of the PVs was observed in a dose-dependent manner when MDP was omitted. The presence of MDP did not interfere with the degradation of the viral genome as confirmed by PCR analysis of irradiated viruses.

Thus, we show for the first time in members of the Picornaviridae family, that the MDP complex effectively uncouples the oxidative damage of proteins from genome degradation during IR-inactivation.

The analysis of residual infectivity and the inactivation rate are important aspects of poliovirus vaccine development. In 1955, Cutter Laboratories released two incompletely inactivated IPV lots that resulted in the death of 10 children and paralysis of 164 vaccinees. This accident led to improved understandings of the non-linear formalin inactivation curves of PV and emphasized the need to ensure that vaccine preparations are void of infectivity. Our pilot study suggested that 40 kGy of gamma radiation was sufficient to inactivate 4 μg of PV, but when the virus sample size was increased to 16 μg, approximately 4 CCID_50_ of residual infectivity was observed. Numerous additional experiments demonstrated that 45 kGy extinguished detectable infectivity in a 16 μg sample. An analysis of the linear region of the inactivation curve shown (Fig 1A) reveals an inactivation equation of y = -8 × 10^7^ln(x) + 3 × 10^8^ with a y-axis intercept at approximately 42.5 kGy which agrees with observed data. The second-order nature of the equation allows improved prediction of the amount of IR required to inactivate 100% of the infectivity. In summary, we show that MDP can protect the immunogenic proteins of PV1-S and PV2-S, and MDP-complexed virus can be completely inactivated by exposure to 45 kGy. Moreover, the method is highly scalable and inactivation is complete within 4–5 h, depending on the specific activity of the radioactive source.

This study demonstrates the *in vivo* efficacy of a whole virus Sabin IPV produced under gamma radiation in the presence of MDP. At higher doses of 1 × and 1/2 ×, IR-inactivated PV-S stimulated neutralizing titers similar to the two control vaccines (IPOL and VeroPol) and all rats seroconverted to at least 1:8 titers. Interestingly, at reduced antigen concentrations (down to 1/64 × dose), IR-inactivated PV2-S stimulated neutralizing titers which were considerably higher than the titers stimulated by VeroPol, a vaccine used in many regions. An unpaired Student’s *t*-test demonstrated that 1/32 × Ir-PV2-S sera were not significantly different in titer to the 1 × IPOL and 1 × VeroPol.

We anticipated that Ir-PV2S would be more immunogenic than Ir-PV1S because PV2 is known to be more immunogenic than PV1. This difference in immunogenicity may partially explain why PV2 was eradicated first. However, we did not anticipate that a 1/32 or 1/64 dose of Ir-PV2-S would stimulate neutralizing titers. The results for both Ir-PV1-S and Ir-PV2-S suggest that the MDP-Ir-inactivation method is more sparing to antigenic epitopes on the exterior of the virus than formalin-inactivation and that potentially more doses could be produced per unit of virus; an important aspect of reducing manufacturing costs and meeting demand shortages.

The Ir-MDP platform of vaccine development represents a rapid, scalable technology to develop vaccines against existing, re-emerging, and new pathogens. The Ir-MDP method may also be applied to inactivate the growing number of modified or attenuated live viral vaccine candidates that are stalled in the approval pathway due to safety concerns related to reactivation of pathogenic phenotypes [22]. Thus, the technology could have far-reaching implications in the world of vaccine development.

Lastly, to maintain a polio-free world after eradication, all nations will need to remain vigilant by continuing surveillance and vaccination for an indeterminate period after eradication has been certified. The use of OPV will be discontinued to prevent vaccine-associated disease caused due to reversion to an infectious form. The use of attenuated Sabin strains in the manufacture of IPV will reduce biohazard risks associated with the production and handling of large quantities of neuropathogenic wild type strains. This novel approach can ensure a safe manufacturing process along with a reduced cost to facilitate affordability in production. After eradication, PV will shift from being a biohazard threat to a biosecurity/bioterrorism threat. Both threat-forms call for continued global immunizations and maintaining large stockpiles of an IPV built on the safest and most cost-effective approach. An IR-inactivated “Salk-Sabin” vaccine as described here would address such a strategic goal.

## Supporting information

S1 Fig Coomassie-stained polyacrylamide gel analysis of PV1-S, PV2-S. Purified viruses were denatured and electrophoresed in an SDS-polyacrylamide gel. Total proteins were stained with Coomassie Brilliant Blue. Migrations of BSA (added as a carrier protein) and the three largest virus structural proteins are indicated as are the molecular weights of the size marker proteins.
(PDF)

S2 Fig Analysis of irradiated PV2-S in presence of DP1 or Mn alone. PV2-S was irradiated with (DP1) or MnCl_2_ (Mn) alone as shown. **A**) Aliquots were analyzed by Western blot demonstrating that the addition of DP1 and Mn alone failed to protect PV2 capsid proteins from oxidative damage beyond 30 kGy doses. **B**) RT-PCR shows that the DP1 and Mn alone do not protect the RNA from damage.
(PDF)

S3 Fig Day 35 neutralizing antibody titer against PV2-S. Groups of 4 or 8 rats were immunized with different human doses of IR-inactivated PV2-S (black triangle) and licensed IPV vaccines, IPOL (Sanofi; blue circle) and VeroPol (Staten Serum Institute; red square) on Days 1 and 21 and serum samples were collected on D35 and D49 for evaluating seroconversion. Log_2_ transformed neutralizing antibody titers for D35 are shown here. Horizontal lines indicate mean neutralization values for each group and each data point represents an individual animal within the group. Ir-PV2-S, gamma irradiated PV2-S. P-values from unpaired one-tailed t-tests comparing Ir-PV2-S to either IPOL or VeroPol are indicated. P-values above 0.05 are not shown.
(PDF)

S1 File ARRIVE guidelines checklist. (DOCX)

S1 Raw images (PDF)
