# Supplementary figures and images for: A novel gamma radiation-inactivated sabin-based polio vaccine
Source: PLoS One. 2020 Jan 30;15(1):e0228006. doi: 10.1371/journal.pone.0228006 (PMC6991977; doi:10.1371/journal.pone.0228006)

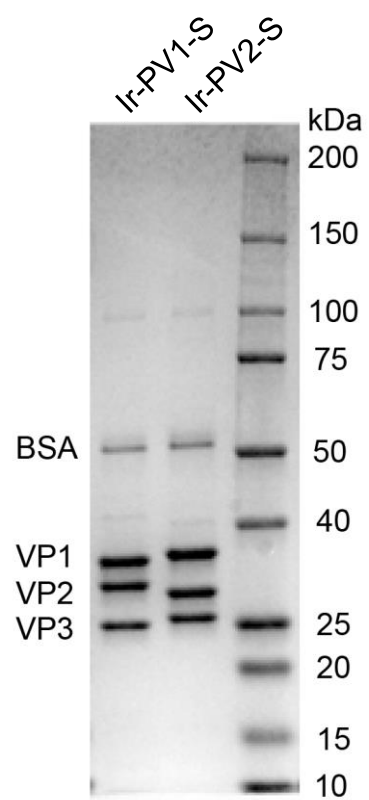

Supplement: S1 Fig — Purified viruses were denatured and electrophoresed in an SDS-polyacrylamide gel. Total proteins were stained with Coomassie Brilliant Blue. Migrations of BSA (added as a carrier protein) and the three largest virus structural proteins are indicated as are the molecular weights of the size marker proteins. (PDF) [file pone.0228006.s001.pdf]

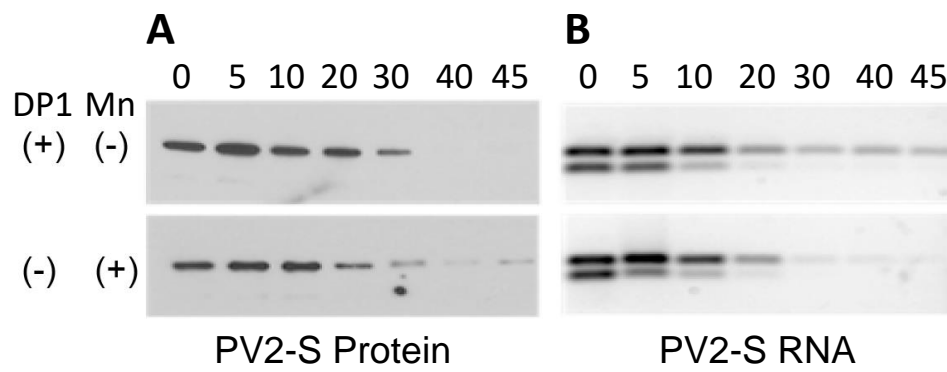

Supplement: S2 Fig — PV2-S was irradiated with (DP1) or MnCl2 (Mn) alone as shown. A) Aliquots were analyzed by Western blot demonstrating that the addition of DP1 and Mn alone failed to protect PV2 capsid proteins from oxidative damage beyond 30 kGy doses. B) RT-PCR shows that the DP1 and Mn alone do not protect the RNA from damage. (PDF) [file pone.0228006.s002.pdf]

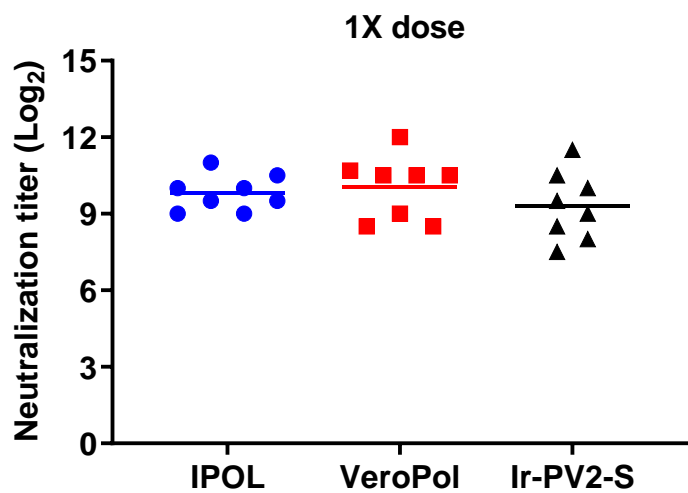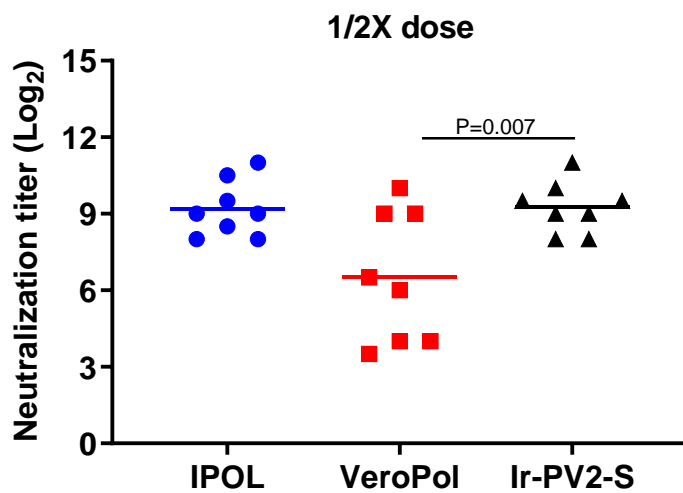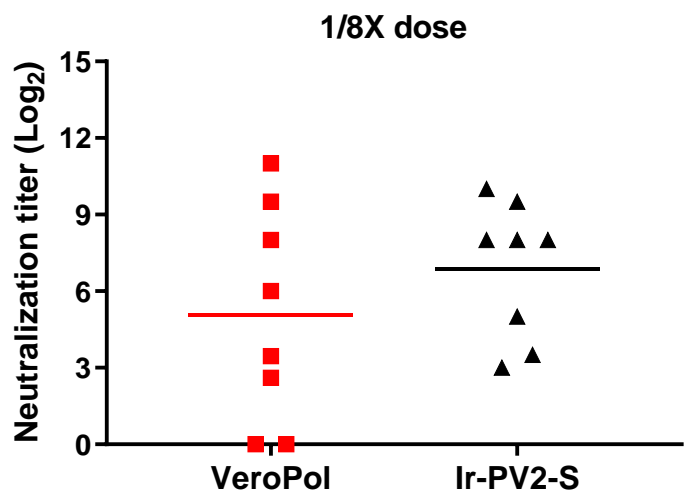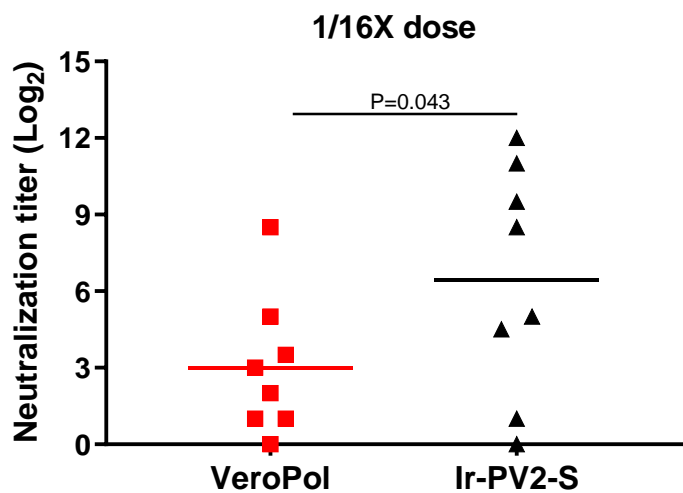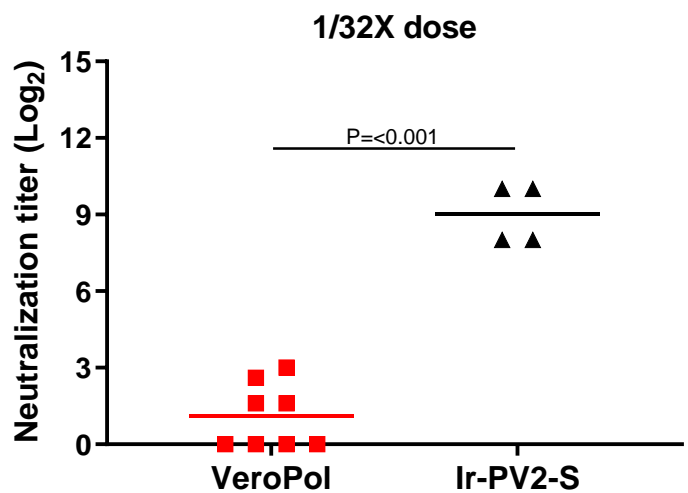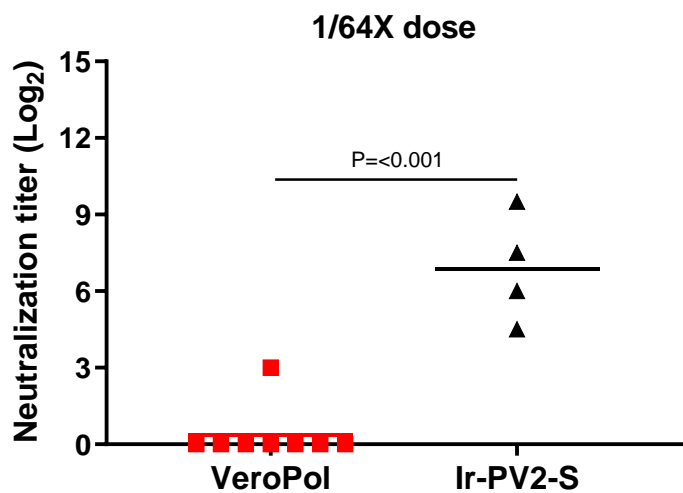

Supplement: S3 Fig — Groups of 4 or 8 rats were immunized with different human doses of IR-inactivated PV2-S (black triangle) and licensed IPV vaccines, IPOL (Sanofi; blue circle) and VeroPol (Staten Serum Institute; red square) on Days 1 and 21 and serum samples were collected on D35 and D49 for evaluating seroconversion. Log2 transformed neutralizing antibody titers for D35 are shown here. Horizontal lines indicate mean neutralization values for each group and each data point represents an individual animal within the group. Ir-PV2-S, gamma irradiated PV2-S. P-values from unpaired one-tailed t-tests comparing Ir-PV2-S to either IPOL or VeroPol are indicated. P-values above 0.05 are not shown. (PDF) [file pone.0228006.s003.pdf]
