# Supplementary material for: A novel gamma radiation-inactivated sabin-based polio vaccine
Source: PLoS One. 2020 Jan 30;15(1):e0228006. doi: 10.1371/journal.pone.0228006 (PMC6991977; doi:10.1371/journal.pone.0228006)
Supplement: S1 Raw images — (PDF) [file pone.0228006.s005.pdf]

Supplementary Information: Raw images of figures showing protein and DNA gels/blots

"A Novel Gamma Radiation-Inactivated Sabin-Based Polio Vaccine"
Tobin, GJ, et al.
PONE-D-19-18205R1

Figure 2A

1 2 3 4 5 6 7 8 9 10 11 12 13 14 15

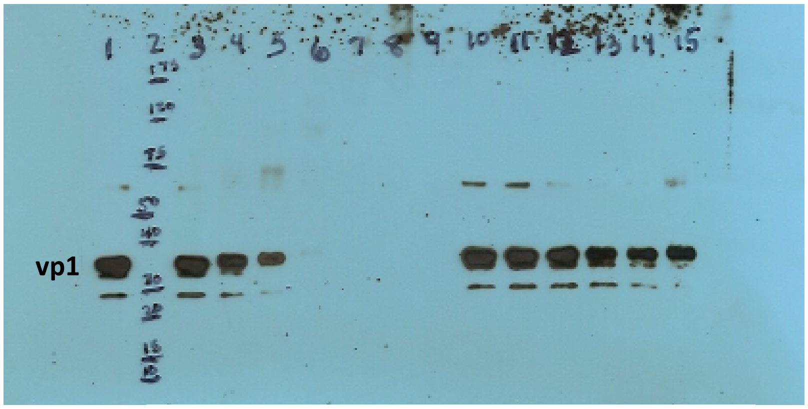

0 5 10 20 30 40 0 5 10 20 30 40 kGy

------No MDP------ -------With MDP------

Figure 2A. Western blot of PV1-S after treatment with gamma irradiation. Prior to irradiation, the virus was complexed with (right side) or without (left side) MDP. The polyclonal antibody used in detection binds primarily to vp1 (see Methods). The blot shows that MDP protects the proteins of PV1-S from damage during ionizing radiation.

Lane	Sample
1.	PV1-S untreated
2.	Protein markers
3.	PV1-S 0 kGy, no MDP
4.	PV1-S 5 kGy, no MDP
5.	PV1-S 10 kGy, no MDP
6.	PV1-S 20 kGy, no MDP
7.	PV1-S 30 kGy, no MDP
8.	PV1-S 40 kGy, no MDP
9.	Empty lane for spacer
10.	PV1-S 0 kGy, with MDP
11.	PV1-S 5 kGy, with MDP
12.	PV1-S 10 kGy, with MDP
13.	PV1-S 20 kGy, with MDP
14.	PV1-S 30 kGy, with MDP
15.	PV1-S 40 kGy, with MDP

Figure 2B

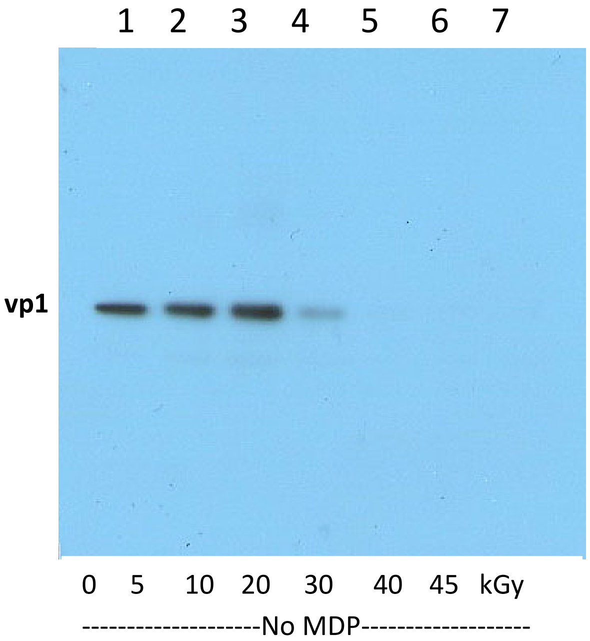

Upper panel (no MDP)

Lane	Sample
1.	PV2-S 0 kGy, no MDP
2.	PV2-S 5 kGy, no MDP
3.	PV2-S 10 kGy, no MDP
4.	PV2-S 20 kGy, no MDP
5.	PV2-S 30 kGy, no MDP
6.	PV2-S 40 kGy, no MDP
7.	PV2-S 45 kGy, no MDP

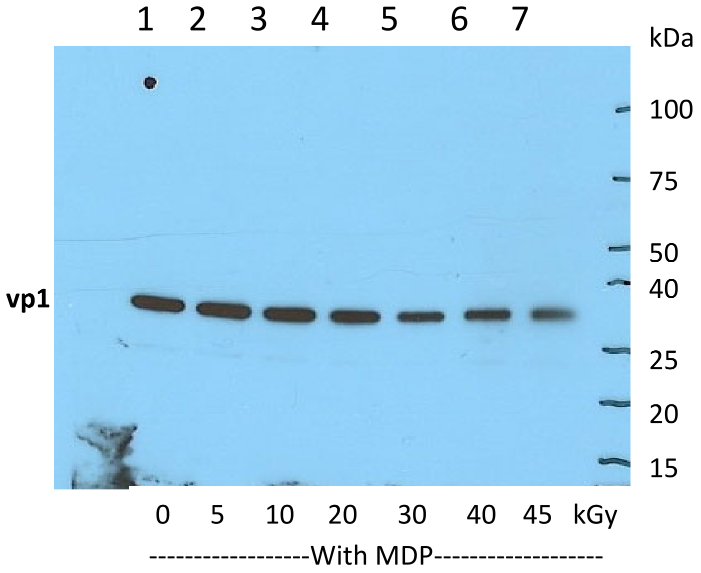

Lower panel (With MDP)

Lane	Sample
1.	PV2-S 0 kGy, with MDP
2.	PV2-S 5 kGy, with MDP
3.	PV2-S 10 kGy, with MDP
4.	PV2-S 20 kGy, with MDP
5.	PV2-S 30 kGy, with MDP
6.	PV2-S 40 kGy, with MDP
7.	PV2-S 45 kGy, with MDP

Figure 2B. Western blot of PV2-S after treatment with gamma irradiation. Prior to irradiation, the virus was complexed with (lower panel) or without (upper panel) MDP. The polyclonal antibody used in detection binds primarily to vp1 (see Methods). The blot shows that the inclusion of MDP protects the proteins from damage during exposure to ionizing radiation.

Figure 2C

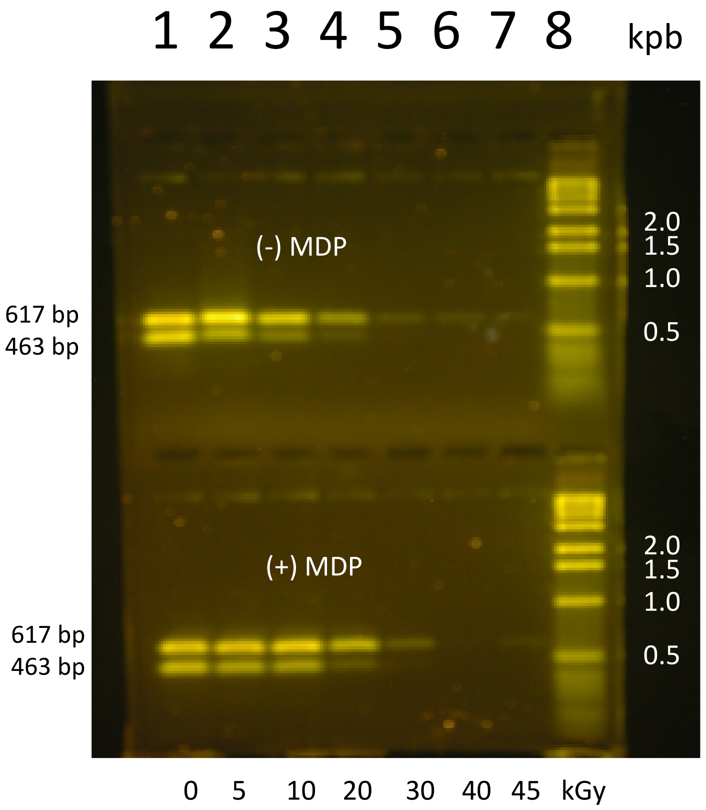

Fig. 2C. RNA damage by gamma irradiation of PV2-S

Top Panel – PV2S irradiated without DP1 peptide and MnCl2

Bottom – PV2S irradiated with both DP1 and MnCl2.

Lane	Sample
1.	0 kGy
2.	5 kGy
3.	10 kGy
4.	20 kGy
5.	30 kGy
6.	40 kGy
7.	45 kGy
8.	DNA size ladder in kilobase pairs

Figure 2C. Analysis of PV2-S RNA damage with increasing doses of gamma irradiation. PV2-S was exposed to varying doses of gamma irradiation without MDP (Top panel) or with MDP (Bottom panel). After irradiation, RNA was purified from virions and cDNA was synthesized using oligo(dT). The cDNA was analyzed by semi-quantitative PCR to amplify and detect a fragment adjacent to the site of cDNA priming (upper band) and a fragment more distal (lower band) (see Methods).

The figure shows that the RNA is fragmented whether or not the virus had been complexed with MDP during irradiation.

Figure S1

1 2 3 4 5 6 7

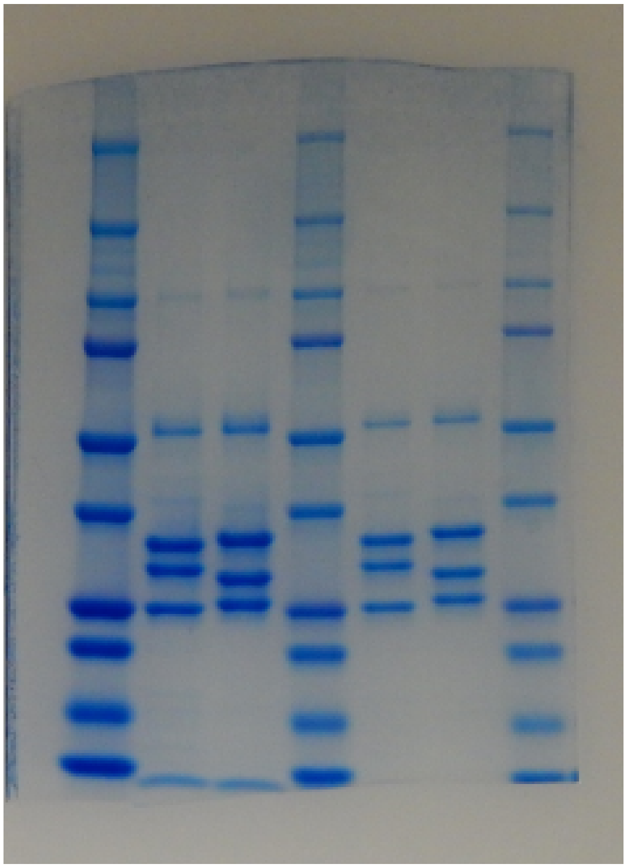

kDa
200
150
100
75
50
40
25
20
15
10

Figure S1
Coomassie-stained protein gel of PV1S and PV2S virions.

Lanes	Samples
1	Protein size markers
2	PV1-S
3	PV2-S
4	Protein size markers
5	PV1-S
6	PV2-S
7	Protein size markers

Numbers to the right of the figure show approximate sizes of the molecular weight marker proteins electrophoresed in Lanes 1, 4, and 7. Lanes 5, 6, and 7 are shown in Figure S1 of the Manuscript

Figure S1. Coomassie-stained protein gel of PV1-S and PV2-S virions. Viruses were propagated in HeLa suspension cultures and purified by sucrose density gradients containing 0.1% BSA as a carrier protein. The figure shows the protein profiles of denatured virus electrophoresed in a 4-20% polyacrylamide gel. Approximately 1 microgram of virus was loaded in Lanes 2 and 3 and 0.5 microgram in Lanes 5 and 6. After electrophoresis, the gel was stained with Coomassie Brilliant Blue. Sizes of the molecular weight marker proteins is shown to the right of the gel image. The protein migrating slightly slower that 50kDa is bovine serum albumin (BSA) which was added to the virus preparation as carrier protein.

Figure S2 – A

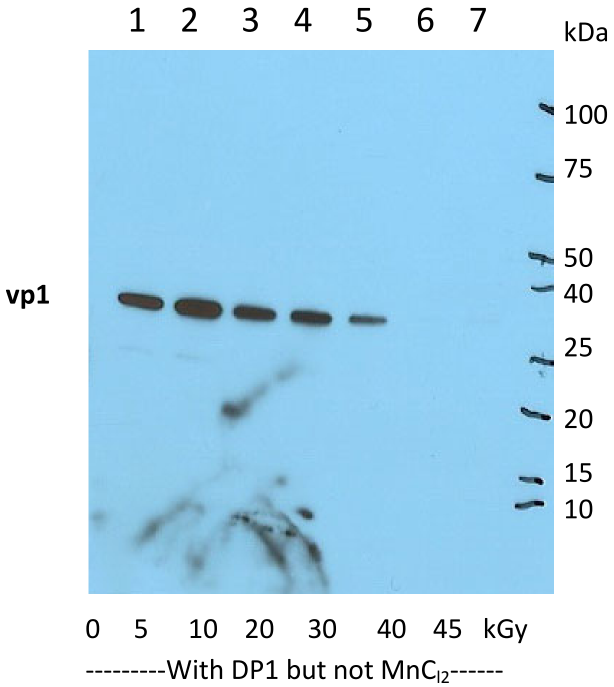

Upper panel (with DP1 alone)

<u>Lane</u> <u>Sample</u>

1. PV2-S 0 kGy, DP1 alone
2. PV2-S 5 kGy, DP1 alone
3. PV2-S 10 kGy, DP1 alone
4. PV2-S 20 kGy, DP1 alone
5. PV2-S 30 kGy, DP1 alone
6. PV2-S 40 kGy, DP1 alone
7. PV2-S 45 kGy, DP1 alone

Lower panel (with MDP)

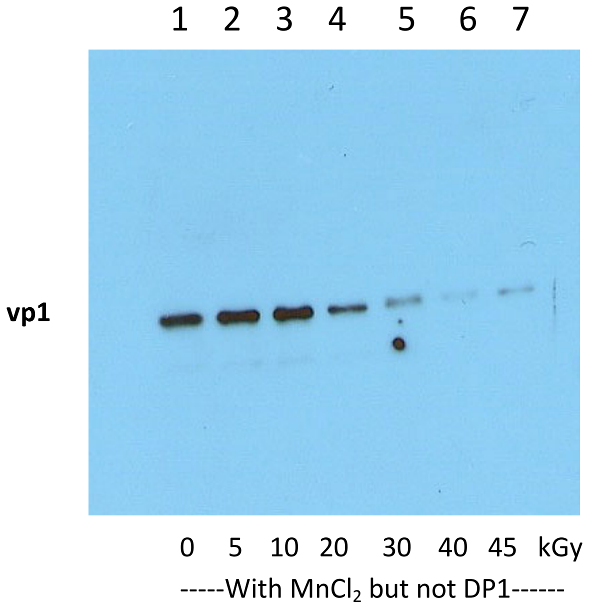

<u>Lane</u> <u>Sample</u>

1. PV2-S 0 kGy, $MnCl_2$ alone
2. PV2-S 5 kGy, $MnCl_2$ alone
3. PV2-S 10 kGy, $MnCl_2$ alone
4. PV2-S 20 kGy, $MnCl_2$ alone
5. PV2-S 30 kGy, $MnCl_2$ alone
6. PV2-S 40 kGy, $MnCl_2$ alone
7. PV2-S 45 kGy, $MnCl_2$ alone

Figure S2-A. Western blot of PV2-S after treatment with gamma irradiation. Prior to irradiation, the virus was complexed with DP1 alone (upper panel) or with $MnCl_2$ alone (lower panel). Following irradiation, the samples were denatured, electrophoresed in a denaturing polyacrylamide gel, and transferred to nitrocellulose for immune-blotting. The antibody used in detection binds primarily to vp1. The numbers to the right of the top panel show migration patterns of molecular weight marker proteins. The blot shows that the inclusion of $MnCl_2$ or DP1 alone does not protect the proteins from damage during exposure to ionizing radiation.

Figure S2-B

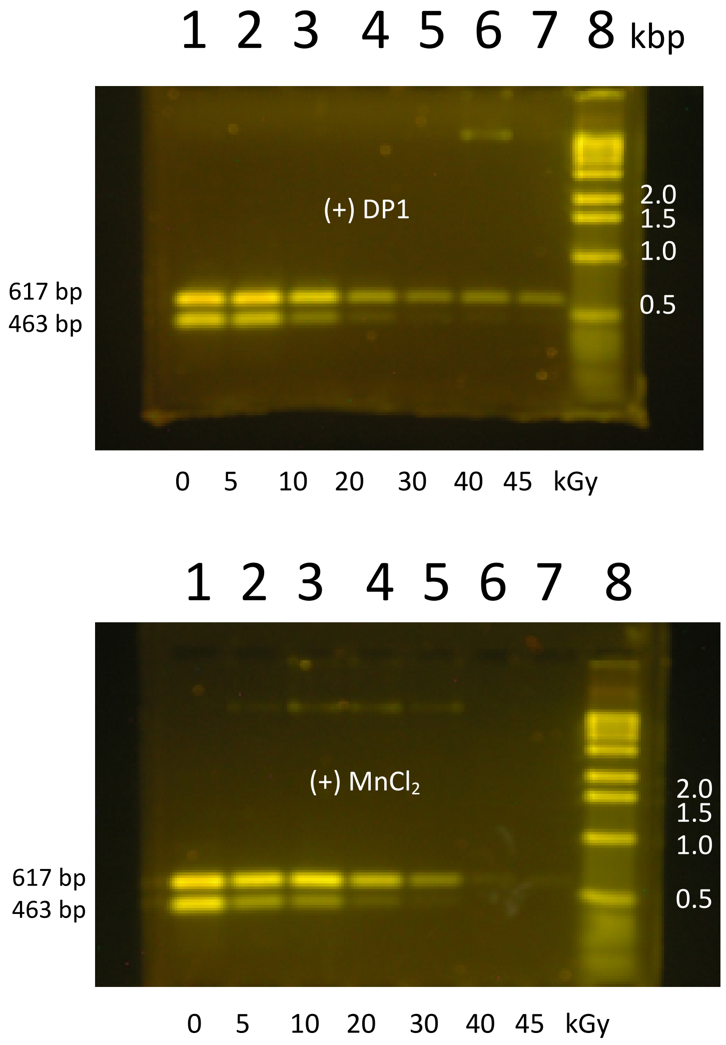

Fig. S2-B. RNA damage by gamma irradiation of PV2-S.
Top – PV2S irradiated with DP1 peptide and without MnCl2
Bottom – PV2S irradiated with MnCl2 and without DP1.

Lanes Samples
1. 0 kGy
2. 5 kGy
3. 10 kGy
4. 20 kGy
5. 30 kGy
6. 40 kGy
7. 45 kGy
8. DNA size ladder in kilobase pairs

Figure S2-B. Analysis of PV2-S RNA damage with increasing doses of gamma irradiation. PV2-S was exposed to varying doses of gamma irradiation without MDP (Top panel) or with MDP (Bottom panel). After irradiation, RNA was purified from virions and cDNA was synthesized using oligo(dT). The cDNA was analyzed by semi-quantitative PCR to amplify and detect a fragment adjacent to the site of cDNA priming (upper band) and a fragment more distal (lower band) (see Methods).
The figure shows that the RNA is fragmented whether or not the virus had been complexed with MDP during irradiation.
